# Chloroquine and hydroxychloroquine-related ocular adverse events in SLE treatment: a real-world disproportionality analysis based on FDA adverse event reporting system (FAERS)

**DOI:** 10.3389/fphar.2025.1498814

**Published:** 2025-06-30

**Authors:** Xiang Li, Si-Qi Zhang, Ke-Rui Wang, Shi-Nan Wu, Meng-Yuan Wang, Cui-Ting Chen, Nuo Dong

**Affiliations:** ^1^ Xiamen Eye Center and Eye Institute of Xiamen University, School of Medicine, Xiamen, China; ^2^ Xiamen Clinical Research Center for Eye Diseases, Xiamen, Fujian, China; ^3^ Xiamen Key Laboratory of Ophthalmology, Xiamen, Fujian, China; ^4^ Fujian Key Laboratory of Corneal & Ocular Surface Diseases, Xiamen, Fujian, China; ^5^ Xiamen Key Laboratory of Corneal & Ocular Surface Diseases, Xiamen, Fujian, China; ^6^ Translational Medicine Institute of Xiamen Eye Center of Xiamen University, Xiamen, Fujian, China; ^7^ Huaxia Eye Hospital of Quanzhou, Quanzhou, Fujian, China; ^8^ Department of Ophthalmology, Affiliated People’s Hospital and Zhenjiang Kangfu Eye Hospital, Zhenjiang College, Zhenjiang, Jiangsu, China; ^9^ Department of Oncology, Xiang’an Hospital of Xiamen University, Xiamen, Fujian, China

**Keywords:** cloroquine, hydroxychloroquine, ocular adverse events, FAERS, systemic lupus erythematosus

## Abstract

**Objective:**

This study aimed to evaluate the risk of adverse events associated with chloroquine (CQ) and hydroxychloroquine (HCQ) in patients with systemic lupus erythematosus (SLE), using data from the U.S. Food and Drug Administration Adverse Event Reporting System (FAERS).

**Methods:**

Disproportionality analysis was conducted using the Reporting Odds Ratio (ROR) to detect potential safety signals. Sensitivity analyses were performed to validate these signals, and the time to onset for each Preferred Term (PT) was assessed.

**Results:**

Between 2004 and 2024, a total of 2,575 adverse event reports related to HCQ or CQ use in patients with SLE were identified in the FAERS database, of which 437 involved ocular adverse events. The most frequently reported ocular conditions were cataract, macular degeneration, and glaucoma. Disproportionality analysis demonstrated strong associations between HCQ/CQ use and retinal degeneration (ROR = 28.5, 95%CI: 19.94–40.74), cystoid macular oedema (ROR = 12.46, 95%CI: 8.01–19.37), and optic atrophy (ROR = 6.55, 95%CI: 3.51–12.19). Sensitivity analyses, conducted after excluding SLE cases, indicated that all but one event (vitreous floaters) remained statistically significant, suggesting that these risks are more likely attributable to HCQ/CQ exposure than to the underlying disease. The time-to-onset analysis showed that cataract had the shortest average onset time (125.5 days), whereas retinal degeneration had the longest (937.5 days).

**Conclusion:**

The extensive clinical use of HCQ and CQ raises significant concerns regarding their ocular safety profile. This study provides real-world pharmacovigilance evidence supporting a substantial risk of ocular adverse events associated with HCQ/CQ use. Further mechanistic and prospective studies are warranted to elucidate the underlying pathophysiological pathways and to confirm these associations.

## Introduction

Systemic lupus erythematosus (SLE) is a chronic autoimmune disorder that affects multiple organs and tissues, including the skin, joints, kidneys, heart, lungs, blood, and nervous system. Its pathogenesis is characterized by immune system abnormalities, wherein the immune response targets normal tissues and organs, leading to inflammation and tissue damage (O’Donnell and Nabel, 2011). In the management of SLE, chloroquine and hydroxychloroquine are extensively employed due to their efficacy in reducing disease activity and preventing disease progression. However, recent in-depth investigations into the safety profiles of these agents have highlighted ocular complications as a significant concern. Notably, systemic administration of these drugs, particularly in the treatment of dermatological conditions, has been associated with a range of serious ocular adverse effects.

Prolonged use of chloroquine and hydroxychloroquine can result in irreversible damage to the retinal pigment epithelium and the photoreceptor complex. This damage typically arises after extended exposure and is associated with the accumulation of melanin within the retinal tissues, particularly when recommended dosages are exceeded or when treatment is prolonged. To facilitate early detection and prevent progression to severe conditions such as bull’s eye maculopathy (Pryor et al., 2022), novel diagnostic and imaging techniques—such as optical coherence tomography (OCT) and wide-field fundus autofluorescence (FAF)—have been increasingly employed.

The study conducted by Radun et al. further underscores the importance of monitoring quantitative autofluorescence (QAF) in patients receiving chloroquine or hydroxychloroquine therapy. Their findings demonstrate a significant increase in QAF values over a 1-year follow-up period, suggesting rapid progression of ocular structural changes and the potential development of bull’s eye maculopathy (Singh et al., 2023).

The Adverse Event Reporting System (FAERS), managed by the United States Food and Drug Administration (FDA), represents the largest spontaneous reporting database, encompassing over 27 million submissions related to adverse events, medication errors, and product quality issues reported to the FDA. This database provides a comprehensive overview of adverse events observed in real-world clinical settings (Hu et al., 2022). The present study aims to evaluate ocular adverse reactions associated with the use of chloroquine and hydroxychloroquine in the treatment of systemic lupus erythematosus, thereby offering clinicians a more robust basis for risk assessment and monitoring recommendations for these medications (Hu et al., 2022).

## Materials and methods

### Data sources

This study was conducted as a retrospective observational pharmacovigilance analysis based on the publicly accessible FAERS database. All methodologies and procedures strictly adhered to the guidelines and standards outlined in the Recommendations for Evaluation and Analysis of Disproportionality in the Use of Safety Signal Detection Based on Individual Case Safety Reports (READUS-PV) (Li et al., 2025b). Given that the FAERS database is publicly available and contains anonymized patient information, informed consent and ethical approval were not required for this study.

FAERS (accessible at https://fis.fda.gov/extensions/FPD-QDE-FAERS/FPD-QDE-FAERS.html) is a publicly available pharmacovigilance database that collects reports of adverse events (AEs), medication errors, and product quality complaints from healthcare professionals worldwide (Long et al., 2024). The database is structured into seven interlinked subfiles, each connected through unique identifiers to form the complete FAERS dataset.

**FIGURE 1:**
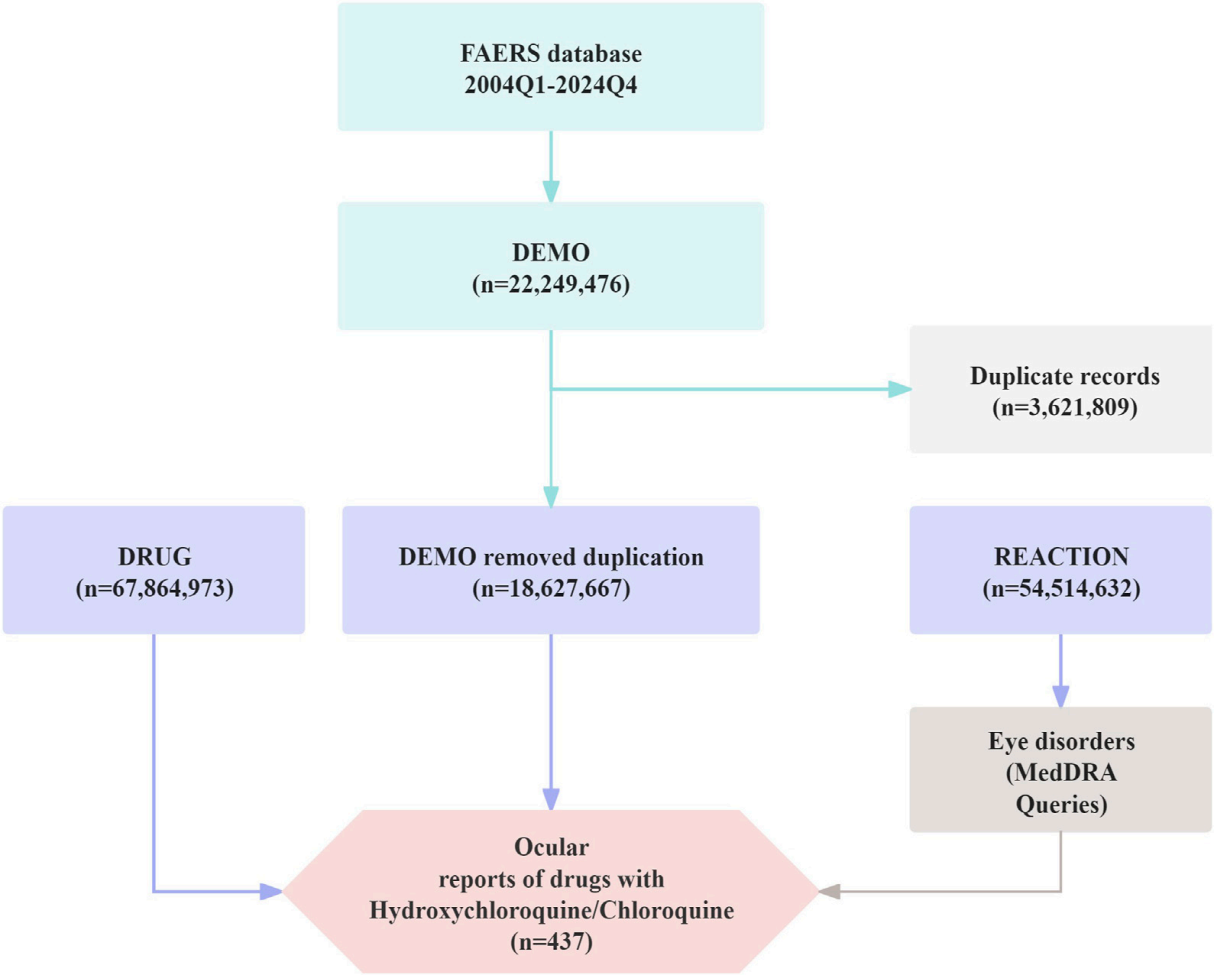
Flow diagram illustrating the selection process of ocular adverse events associated with Chloroquine and Hydroxychloroquine from the FAERS database.

To ensure the accuracy and reliability of the data, only cases reported by healthcare professionals—specifically physicians and pharmacists (coded as MD and PH)—were included in the analysis (Li et al., 2025a). Between January 2004 and December 2024, FAERS recorded a total of 22,249,476 initial reports; after deduplication, 18,627,667 valid records were retained for analysis. Detailed information is presented in Figure 1.

### Data processing

To obtain reports of adverse events associated with hydroxychloroquine (HCQ) or chloroquine (CQ) treatment, relevant records were identified from the DRUG file using the generic and brand names approved by the FDA. This study specifically focused on adverse events classified under the “Eye Disorders” System Organ Class (SOC code: 10015919) in cases where HCQ or CQ was used for the treatment of systemic lupus erythematosus, with the aim of detecting potential ocular toxicity induced by these drugs. In addition, the onset time of ocular adverse events was assessed, defined as the interval between the initiation of HCQ or CQ therapy and the occurrence of the adverse event. Only cases with an onset time greater than 0 days were included in the analysis (Li et al., 2025c); reports containing erroneous dates (e.g., drug initiation after event occurrence) or missing date information were excluded. The control group comprised individuals exposed to non-target drugs, whereas the experimental group consisted of individuals exposed to HCQ or CQ.

### Data analysis

Signal detection in this study was performed using the disproportionality analysis method of reporting odds ratio (ROR). This method is based on a 2 × 2 contingency table (see Table 1), and the ROR was calculated using the following formula: ROR = $\frac{a/c}{b/d}=\frac{\text{ad}}{bc}$ , 95%CI = $e^{\ln\left(ROR\right)\pm 1.96\sqrt{\frac{1}{a}+\frac{1}{b}+\frac{1}{c}+\frac{1}{d}}}$ (Luo et al., 2025). A positive signal was considered present when the number of co-reported cases (a) between a specific drug and an adverse event was ≥3, and the lower bound of the 95% confidence interval (CI) exceeded 1, indicating a potential association between the drug and the adverse event (Liu et al., 2025). Categorical variables were summarized as frequencies and percentages. All statistical analyses were performed using R software (version 4.3.2) and Microsoft Excel (2021).

**TABLE 1:** Proportional imbalance method four-grid table.

Item	Reports with the target AEs	All other AEs	Total
Reports with the target drug	a	b	a+b
All other drugs	c	d	c+d
Total	a+c	b+d	a+b+c+d

Abbreviation: AEs, Adverse Events.

## Results

### Descriptive analysis

**FIGURE 2:**
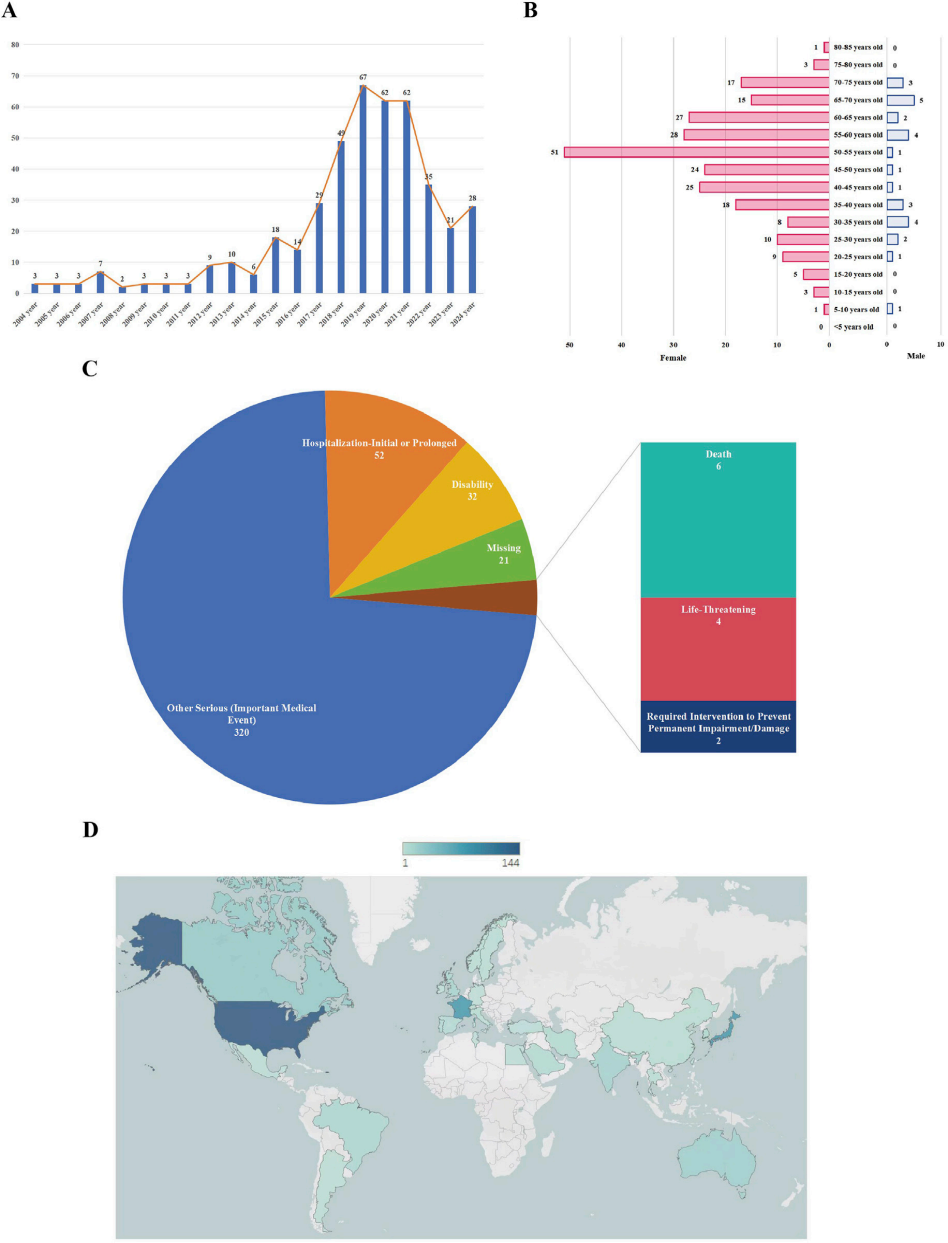
The characteristics of chloroquine- and hydroxychloroquine-related ocular adverse events in the treatment of systemic lupus erythematosus. **(A)** Annual distribution of ocular adverse event reports from 2004 to 2022; **(B)** Age and sex distribution of patients with reported ocular adverse events; **(C)** Proportions of different serious outcomes; **(D)** Geographic distribution of reported cases by country.

From 2004 to 2022, the number of adverse event reports related to HCQ or CQ exhibited a continuous upward trend (Figure 2A). By December 2024, a total of 2,575 patients had reported adverse events associated with HCQ or CQ treatment for systemic lupus erythematosus, among which 437 cases were classified as ocular adverse events. The mean age of these patients was 49.45 ± 15.07 years, as detailed in Table 1. In terms of sex distribution, a higher incidence of ocular adverse events was observed among female patients (n = 271, 62.01%) compared with male patients (n = 30, 6.86%). Notably, the age distribution of patients approximated a normal curve, characterized by an initial increase followed by a subsequent decline, with the highest number of reports observed in the 50–55-year age group (Figure 2B). Hospitalization (n = 52, 11.90%) and other serious outcomes, such as important medical events (n = 320, 73.23%), were the most frequently reported serious adverse outcomes (Figure 2C). The geographic distribution of reported cases across countries is shown in Figure 2D. Additionally, individual dosage information for each patient was collected and is presented in Supplementary Table S1.

### The spectrum of ocular adverse effects at the PT level

**FIGURE 3:**
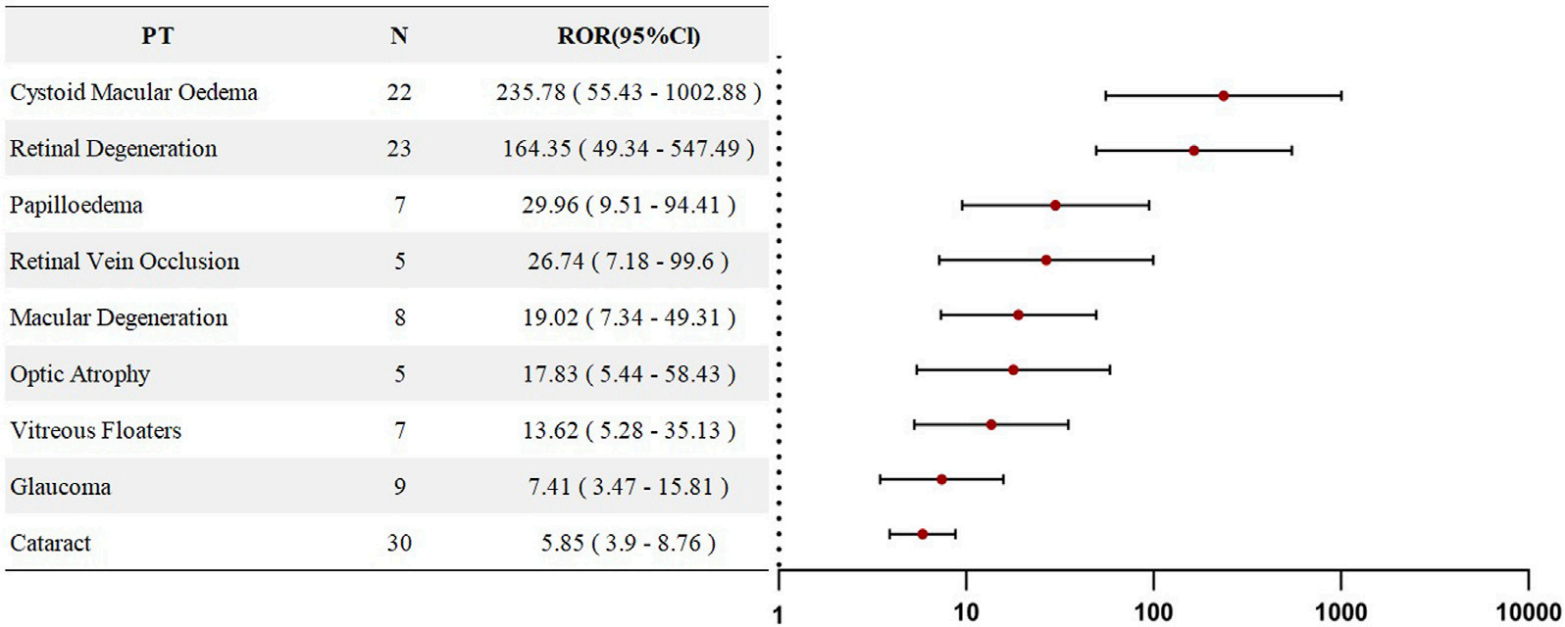
Forest plots of PT-level RORs for Chloroquine- and Hydroxychloroquine-related ocular adverse events in SLE therapy.

An analysis of adverse events associated with HCQ or CQ was conducted at the Preferred Term (PT) level. The three most frequently reported PTs were cataract (n = 78), macular degeneration (n = 42), and glaucoma (n = 33). Disproportionality analysis of these PTs revealed eight signals with positive associations (ROR >1). All PTs listed in Figure 3 demonstrated statistical significance and are ranked in descending order according to their ROR values. The top three adverse events were retinal degeneration (ROR = 28.5), cystoid macular oedema (ROR = 12.46), and optic atrophy (ROR = 6.55), indicating a strong statistical association between HCQ/CQ use and ocular disorders, particularly retinal diseases. Detailed information is presented in Figure 3.

### Sensitivity analysis

**FIGURE 4:**
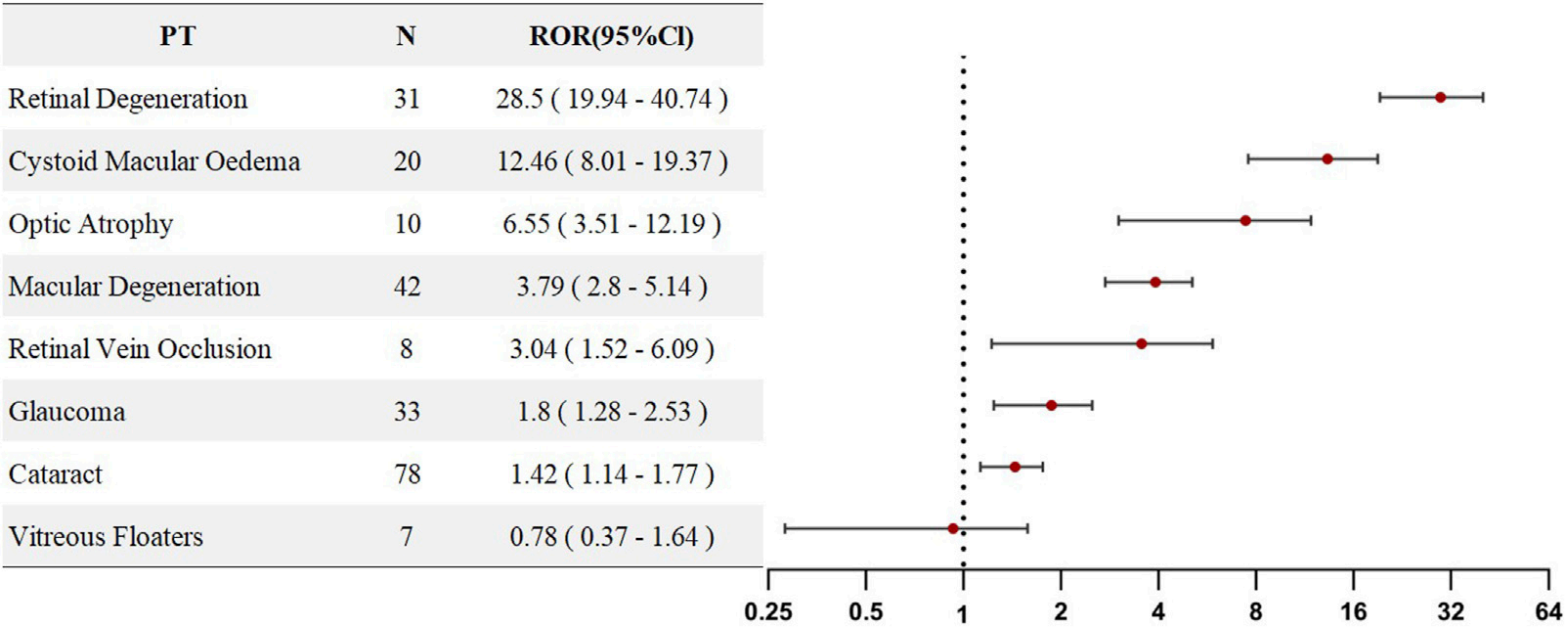
PT-level ROR forest plots for Chloroquine- and Hydroxychloroquine-related ocular AEs excluding SLE patients.

Given that SLE itself carries an inherent risk of vascular retinal diseases, a sensitivity analysis was performed by excluding all individuals who used HCQ or CQ for the treatment of SLE. This analysis aimed to eliminate the potential confounding effect of SLE on the study outcomes. Upon reanalysis, we found that, in the sensitivity analysis, all PTs—except for vitreous floaters (ROR = 0.78)—still exhibited ROR values greater than 1, indicating that SLE did not confound the associations observed for the remaining seven PTs. Rather, these risks appeared to be directly attributable to HCQ/CQ exposure. Detailed results are presented in Figure 4.

### Time-to-onset analysis

Further analysis was conducted to assess the onset time for individuals corresponding to the seven PTs that remained positive in the sensitivity analysis.

**FIGURE 5:**
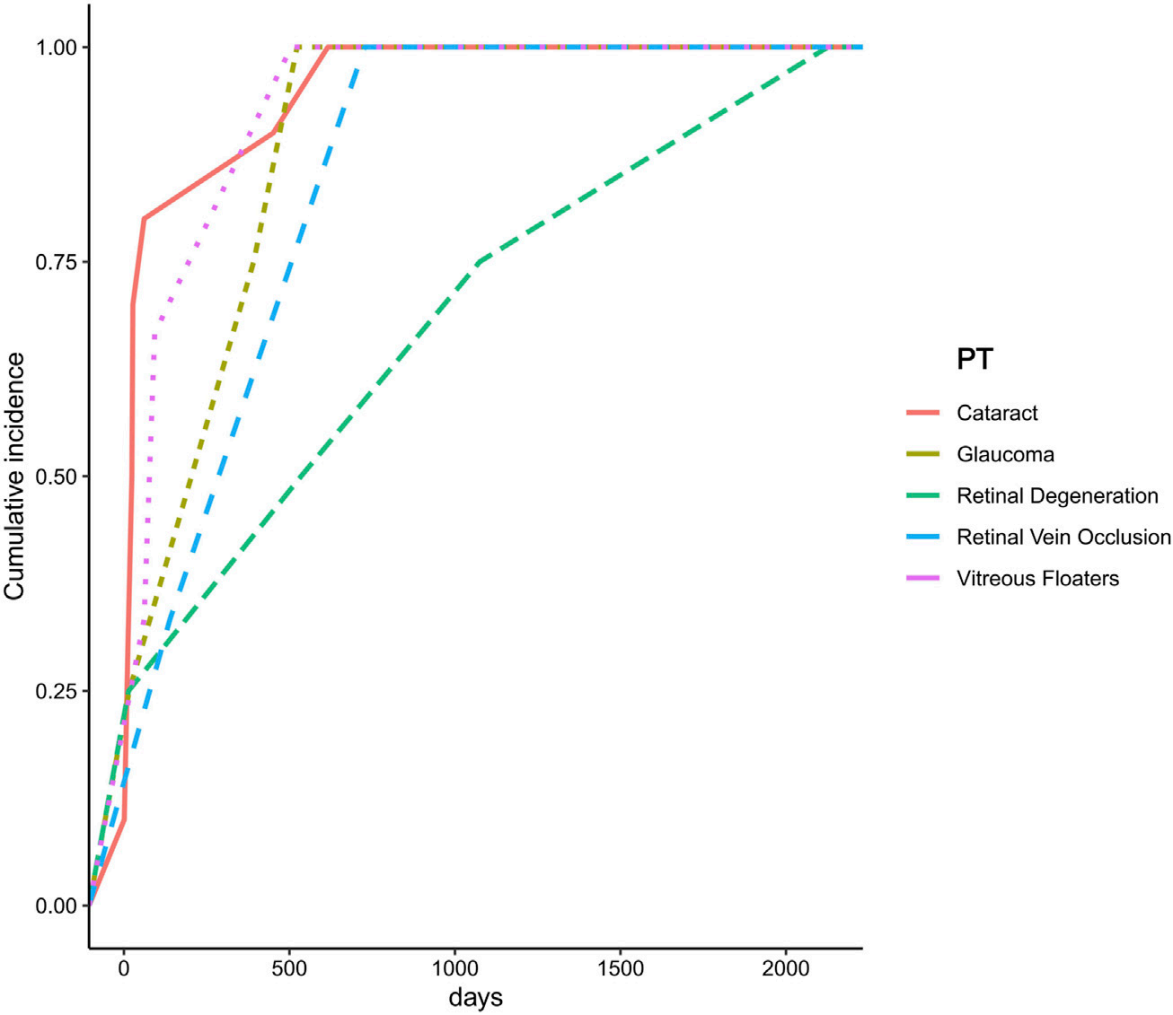
Time to onset of ocular adverse events associated with Chloroquine and Hydroxychloroquine.

Due to missing data, onset time information was available for only five PTs. The results indicated that cataract had the shortest mean onset time, averaging 125.5 days, whereas retinal degeneration exhibited the longest mean onset time, with an average of 937.5 days (Figure 5).

## Discussion

This study, based on real-world data from the FAERS database, comprehensively evaluated the post-marketing safety of HCQ and chloroquine CQ in the treatment of SLE, providing updated insights into ocular adverse events associated with these agents. Previous clinical trials and literature reviews have consistently demonstrated that the use of HCQ or CQ in SLE significantly increases the risk of ocular adverse events. Building upon these findings, the present study systematically identified and characterized the full spectrum of HCQ/CQ-associated ocular adverse events to date, thereby addressing a critical gap in real-world evidence in this field.

SLE is a chronic autoimmune disease characterized by the production of autoantibodies against nuclear and cytoplasmic antigens. It can affect multiple organ systems, presenting with a wide range of clinical manifestations and immunological abnormalities, and typically follows a relapsing–remitting course (Borchers et al., 2010). SLE affects individuals across all races, sexes, and age groups, but it exhibits a higher incidence among African American and Afro-Caribbean populations, with a strong predilection for women between 30 and 40 years of age (Costedoat-Chalumeau et al., 2015). In the present study, baseline demographic characteristics (Table 2) revealed that ocular adverse events were significantly more frequent among female patients than male patients (271 cases vs. 30 cases) and that middle-aged patients (18–64 years, n = 220, 50.34%) were more prone to ocular adverse events compared to elderly patients (>65 years, n = 44, 10.07%). These findings are consistent with the known epidemiological patterns of SLE.

**TABLE 2:** Population baseline information.

Characteristics	HCQ/CQ induced eye AES(N = 437)	HCQ/CQ induced overall AES(N = 2575)
Gender,n (%)		
Female	271 (62.01)	1757 (68.23)
Male	30 (6.86)	228 (8.85)
Unknown	136 (31.12)	590 (22.91)
Weight (kg),n (%)		
<50	25 (5.72)	98 (3.81)
50∼100	1 (0.23)	27 (1.05)
>100	82 (18.76)	288 (11.18)
Unknown	329 (75.29)	2162 (83.96)
Age (years),n (%)		
<18	16 (3.66)	119 (4.62)
18∼64.9	220 (50.34)	1318 (51.18)
65∼85	44 (10.07)	212 (8.23)
>85	0 (0.00)	1 (0.04)
Unknown	157 (35.93)	925 (35.92)
Occupation of reporters,n (%)		
Consumer (CN)	105 (24.03)	367 (14.25)
Physician (MD)	132 (30.21)	814 (31.61)
Pharmacist (PH)	6 (1.37)	60 (2.33)
Health-Professional (HP)	86 (19.68)	700 (27.18)
Other health-professional (OT)	94 (21.51)	540 (20.97)
Unknown	14 (3.20)	94 (3.65)
Reported countries,n(%)		
US	144	1015
Non-US	293	1560
Outcomes,n(%)		
Death (DE)	6 (1.37)	136 (5.28)
Disability (DS)	32 (7.32)	55 (2.14)
Hospitalization (HO)	52 (11.90)	646 (25.09)
Life-Threatening (LT)	4 (0.92)	122 (4.74)
Other serious (Important medical event)(OT)	320 (73.23)	1363 (52.93)
Required intervention to prevent permanent impairment/damage (RI)	2 (0.46)	6 (0.23)
Congenital Anomaly (CA)	0 (0.00)	16 (0.62)
Unknown	21 (4.81)	231 (8.97)

Notes: Continuous numerical variables are expressed as mean ± standard deviation, and categorical variables are presented as n (%).

HCQ and CQ are cornerstone therapies for the treatment of SLE. The use of HCQ and CQ in the management of SLE and other rheumatic diseases has spanned more than 5 decades. Generally, these agents are well tolerated, and treatment discontinuation due to systemic adverse events is relatively uncommon. However, both drugs have been associated with irreversible retinal toxicity, and recent studies suggest that such toxicity may not be as rare as previously believed (Rosenthal et al., 1978).

In our database, a total of 224 cases of retinal-related adverse events were identified. The three most frequently reported conditions were retinal degeneration (n = 31, ROR = 28.5), retinal vein occlusion (n = 8, ROR = 3.04), and optic atrophy (n = 10, ROR = 6.55). Although the precise mechanisms by which HCQ and CQ induce retinal changes remain incompletely understood, it is known that both drugs extensively accumulate in ocular pigmented tissues, particularly within the retinal pigment epithelium (RPE), where they bind tightly to melanin. Importantly, these agents can persist in ocular tissues for extended periods even after discontinuation (Mao et al., 2024).

Histopathological studies of advanced chloroquine retinopathy in humans have demonstrated damage to both rod and cone photoreceptors, with relative preservation of central macular cones (Bernstein and Ginsberg, 1964; Wetterholm and Winter 1964), a finding that explains the clinical manifestation of bull’s eye maculopathy observed on fundoscopic examination. Narrowing of the retinal arterioles is thought to represent a secondary phenomenon following widespread retinal injury, while pigmentary changes may result from the migration of RPE cells. This has been confirmed by the presence of pigment-laden cells within the outer nuclear and outer plexiform layers, suggesting that RPE metabolic dysfunction occurs early in the disease process (Wetterholm and Winter 1964). Impairment of the RPE’s ability to phagocytose photoreceptor outer segments leads to RPE degeneration and subsequent photoreceptor loss (Bernstein and Ginsberg, 1964). These retinal changes are typically irreversible in clinical settings. Although HCQ and CQ are generally well tolerated, updated 2016 guidelines recommend that the maximum daily dose should not exceed 5 mg/kg of actual body weight, as excessive dosing and long-term therapy are considered major risk factors for retinal toxicity (Marmor et al., 2016).

A considerable number of adverse events involving the macular region were also observed, specifically including macular degeneration (n = 42, ROR = 3.79) and cystoid macular oedema (n = 20, ROR = 12.46). These findings suggest a potential association between HCQ or CQ exposure and the development of macular diseases. Previous studies have demonstrated that treatment of ARPE-19 cells with chloroquine induces lysosomal enlargement and intracellular lipid accumulation, implicating lysosomal dysfunction as a potential pathogenic mechanism in macular injury. Additionally, chloroquine has been reported to promote intracellular vesicle formation and disrupt autophagy pathways. Nevertheless, the exact mechanisms underlying the association between HCQ/CQ use and macular diseases remain incompletely understood and warrant further investigation (Chen et al., 2011).

Some of these adverse events have been previously documented in the drug labeling information. However, through database mining, we also identified several adverse events not explicitly listed in the prescribing information, such as cataract (n = 25, ROR = 4.82) and glaucoma (n = 8, ROR = 7.40). All adverse events selected for analysis demonstrated statistical significance (ROR >1 and a ≥3). Following the sensitivity analysis, vitreous floaters yielded a negative result, suggesting that this outcome may be more attributable to the underlying SLE pathology rather than to HCQ or CQ exposure. The specific impact of HCQ or CQ on these newly identified adverse events, as well as the underlying pathogenic mechanisms, remains incompletely understood and warrants further clinical and basic research.

In this study, an onset time analysis was performed for the seven ocular adverse events (PTs) that remained positive in the sensitivity analysis. Due to missing data, onset time information was available for only five PTs. The results revealed significant differences in the time to onset among different types of ocular adverse events following initial drug exposure. Cataract exhibited the shortest mean onset time, averaging 125.5 days, suggesting that the lens may be particularly sensitive to the pharmacological effects of chloroquine and HCQ, with damage manifesting within a relatively short period. However, the current evidence regarding the association between HCQ use and cataract development remains inconclusive. A retrospective cohort study conducted in patients with rheumatoid arthritis found no significant association between HCQ use and cataract formation (adjusted hazard ratio, 1.17; 95% CI: 0.86–1.59; P > 0.05) (Zhang et al., 2023). Therefore, the rapid onset of cataracts observed in this study may be influenced by underlying disease conditions, concomitant medication use (e.g., corticosteroids), or other environmental factors, and warrants further investigation. In contrast, retinal degeneration exhibited the longest mean onset time, averaging 937.5 days, suggesting that retinal damage represents a slow and progressive accumulation process. This finding is consistent with previous reports characterizing hydroxychloroquine-induced retinal toxicity. Studies have shown that HCQ accumulates over time within RPE cells, leading to cellular dysfunction and subsequent photoreceptor damage (Jorge et al., 2018). Moreover, the risk of HCQ-induced retinal toxicity is closely associated with cumulative dose and duration of therapy. The American Academy of Ophthalmology (AAO) recommends initiating retinal toxicity screening after 5 years of HCQ use in patients without additional risk factors (Marmor et al., 2016).

### Limitations

Although this study leveraged a real-world data mining approach based on the FAERS database, several inherent limitations common to all pharmacovigilance databases must be acknowledged. First, issues such as false reports, underreporting, reporting errors, incomplete information, and reporting delays may occur, introducing unavoidable biases. Second, the FAERS database includes only reported adverse event cases (Sakaeda et al., 2013); due to the lack of denominator data (i.e., the total number of patients exposed to HCQ or CQ), the true incidence of HCQ/CQ-related ocular adverse events could not be determined. Third, the association analyses conducted in this study provide only statistical correlations and do not establish definitive causal relationships (Godfrey et al., 2025). Fourth, many adverse event reports within the FAERS database are submitted by non-medical personnel, potentially resulting in improper use of medical terminology or inconsistencies with established medical definitions. For example, retinal vascular occlusion or retinal vein occlusion (RVO) is generally associated with SLE itself (Hsu et al., 2020). Although our database analysis revealed differing trends, it is possible that some of these events were incorrectly reported as adverse reactions related to CQ/HCQ by non-professionals. Additionally, the onset timing of retinal lesions observed in our study differs from the timing of CQ/HCQ-associated retinopathy previously reported in the literature (Dammacco, 2018). This discrepancy may similarly stem from incomplete or inaccurate documentation by non-medical reporters in the FAERS database. Therefore, the interpretation of our findings should be approached with caution.

To address the aforementioned limitations, a more comprehensive approach is required. First, rigorous validation and verification of reported cases through cross-referencing with other medical records should be conducted to ensure data accuracy and reliability. Second, the integration of multiple data sources—including international pharmacovigilance databases, hospital records, and individual health records—would provide a broader and more diversified perspective. Furthermore, combining prospective clinical studies with retrospective data analyses could enable a more balanced and comprehensive assessment of drug-related adverse effects and their epidemiological characteristics. This multilayered strategy would enhance research quality and objectivity, thereby allowing for a more accurate evaluation of the risk–benefit profile of the drugs.

## Conclusion

The widespread use of HCQ and CQ has raised concerns regarding their safety, particularly the risk of ocular AEs. Although spontaneous reporting systems have certain inherent limitations, they remain an important tool for identifying rare adverse events. Based on real-world data from the FAERS database, this study systematically evaluated the risk of ocular adverse events associated with HCQ and CQ. The results demonstrated that most findings were consistent with previous clinical studies and information provided in the prescribing information. In addition, several potential new and unexpected adverse event signals were identified, offering valuable insights for clinical risk monitoring and management.

## Data Availability

The raw data supporting the conclusions of this article will be made available by the authors, without undue reservation.

## Abbreviations

SLE, Systemic Lupus Erythematosus; OCT, Optical Coherence Tomography; FAF, Fundus Autofluorescence; QAF, Quantitative Autofluorescence; FAERS, FDA Adverse Event Reporting System; FDA, The United States Food and Drug Administration; READUS-PV, Recommendations for Evaluation and Analysis of Disproportionality in the Use of Safety Signal Detection Based on Individual Case Safety Reports; AEs, Adverse Events; HCQ, Hydroxychloroquine; CQ, Chloroquine; ROR, Ratio of Odds Ratios; CI, Confidence Interval; PT, Preferred Term; RPE, Retinal Pigment Epithelium; AAO, The American Academy of Ophthalmology.
